# A comparison of treatment with adefovir and entecavir for chronic hepatitis B in China: The 2-year results of a prospective study: Adefovir versus Entercavir for Chronic Hepatitis B

**Published:** 2011-01-01

**Authors:** En Qiang Chen, Tao You Zhou, Li Liu, Cong Liu, Ming Lei, Hong Tang

**Affiliations:** 1 Center of Infectious Diseases, West China Hospital, Sichuan University, Chengdu, Sichuan, People’s Republic of China; 2 Division of Infectious Diseases, State Key Laboratory of Biotherapy, Sichuan University, Chengdu, Sichuan, People’s Republic of China

**Keywords:** Adefovir, Entecavir, Chronic hepatitis B, Comparison

## Abstract

**Background:**

The reduction of hepatitis B virus replication to minimal levels is emerging as key therapeutic goal in chronic hepatitis B (CHB).

**Objectives:**

This study aimed to evaluate and compare the efficacies of adefovir (ADV) and entecavir (ETV) in CHB.

**Patients and Methods:**

In this prospective study, 100 naïve patients were assigned to treatment with ADV (33 HBeAg-positive and 19 HBeAg-negative patients) or ETV (32 HBeAg-positive and 16 HBeAg-negative patients). The primary efficacy outcome was ALT normalization, reduction in HBV DNA, and seroconversion of HBeAg. Second efficacy outcomes included resistance and safety. Comparisons of quantitative and qualitative variables between groups were analyzed by student t-test and chi-square test (or Fisher's exact test), respectively.

**Results:**

Among HBeAg-positive patients, ETV was superior to ADV with respect to mean reduction in HBV DNA (-7.5 versus -6.3, respectively, at Month 24, p = 0.003) and the percentage of those with HBV DNA < 103 copies/mL at Month 24 [96.9% (31/32) vs. 69.7% (23/33), respectively, p = 0.002] and < 300 copies/mL at Month 24 [84.4% (27/32) vs. 54.5% (18/33), respectively, p = 0.004]. But, the rates of ALT normalization and HBeAg seroconversion between the groups were similar [87.9% (29/33) vs. 96.9% (31/32), respectively, p=0.355; and 24.2% (8/33) vs. 25.0% (8/32), respectively, p = 0.943]. In HBeAg-negative patients who received ETV or ADV, the reduction in HBV DNA (-6.8 versus -5.9, respectively, p = 0.192), percentage of ALT normalization [100% (16/16) vs. 78.9% (15/19), respectively, p=0.109], HBV DNA < 103 copies/mL [100% (16/16) vs. 89.5% (17/19), respectively, p = 0.489], and HBV DNA < 300 copies/mL [100% (16/16) vs. 84.2% (16/19), respectively, p = 0.234] were similar. No ETV- or ADV-associated mutations were observed, and both agents were well tolerated.

**Conclusions:**

ETV and ADV are effective therapies for CHB. In HBeAg-positive patients, the efficacy of ETV is significantly superior to that of ADV, and in HBeAg-negative patients, the agents effect similar biochemical and virological responses.

## Background

More than 400 million individuals worldwide are chronically infected with hepatitis B virus [1], and chronic hepatitis B (CHB) can progress to cirrhosis, hepatocellular carcinoma (HCC), and death [2]. Antiviral therapy is used in CHB to minimize liver damage and limit disease progression [3]. The most significant risk factor for the development of cirrhosis or HCC is serum HBV DNA level [4][5], and it appears that the sustained suppression of serum HBV DNA replication is essential for impeding or reversing disease progression. Nucleoside and nucleotide analogs can function as antiviral agents by inhibiting HBV replication and competing with the natural nucleotide substrate of DNA polymerase, thereby terminating the synthesis of viral DNA [6].In the last decade, 4 nucleoside and nucleotide analogs (lamivudine, adefovir (ADV), entecavir (ETV), and telbivudine) were approved for the treatment of CHB in China [7][8]. These agents vary with respect to antiviral and clinical efficacy, resistance profiles, tolerability, and safety. With the introduction of the concept of long-term management for CHB [9][10], the clinical challenge is to determine how to use available agents most effectively to obtain consistent, profound, and long-lasting HBV suppression with good safety and convenience in a variety of health care settings [11]. According to a World Health Organization (WHO) estimate, one-third of CHB patients reside in China. Recently, some studies have reported that the race or ethnicity of patients affects the efficacy of anti-HBV treatment [12][13][14]. Unfortunately, few studies have been performed in Chinese CHB patients. It is unknown whether treatment with nucleoside analogs in Chinese CHB patients differs compared with patients from other regions.ADV is an oral acyclic nucleotide analog with potent and specific effects against HBV infection. With the availability of tenofovir [15], ADV has begun to be withdrawn from certain regions. Due to its lengthy time to market and high cost, however, the likelihood of obtaining tenofovir remains distant for the majority of CHB patients. In China, ADV remains widely used as a first-line antiviral agent. Recently, HBeAg-negative status and low baseline serum HBV DNA levels have been reported to be associated with virological response in ADV-treated CHB patients [16].

## Objectives

This study compared the efficacy and safety of long-term treatment with ADV and ETV in HBeAg-positive and HBeAg-negative CHB patients.

## Patients and Methods

### Patients

Outpatients from West China Hospital with chronic hepatitis B, aged 18 years or older, were screened and included in this study, per the following criteria: positive reading for hepatitis B surface antigen (HBsAg) for at least 6 months; serum HBV DNA load above 1000 copies/ml at baseline; and meeting the general indications for antiviral therapy, as recommended by the Chinese Society of Hepatology.The exclusion criteria were: presence of serum antibodies against hepatitis C virus (HCV) or human immunodeficiency virus (HIV); a course of preantiviral agent therapy (nucleoside analogs or interferon) for more than 3 months; breastfeeding, pregnancy, or inadequate contraceptive measures; substance abuse in the previous 2 years; other acquired or inherited causes of liver disease; serious concomitant disease; and advanced liver disease (including decompensated cirrhosis with ascites, severe hepatitis, and hepatic carcinoma).

### Study design

This prospective, controlled study evaluated and compared the efficacy of ADV and ETV in Chinese CHB patients. Patients were administered 10 mg ADV (GlaxoSmithKline) or 0.5 mg ETV (Bristol-Myers Squibb) daily according to individual choice, and they were followed up in the outpatient clinic of West China Hospital. Clinical data were collected at baseline and every 3 months after treatment. The primary efficacy outcome was ALT normalization, reduction in HBV DNA, and seroconversion of HBeAg. Second efficacy outcomes included resistance and safety.This study was approved by West China Hospital's institutional review board and was conducted per the 1975 Declaration of Helsinki. All patients signed informed consent forms before their inclusion in this study.

### Serum assay

HBeAg and serum HBV DNA were measured by ELISA (Intec Stone, China) and the PCR-based Cobas Amplicor HBV Monitor Test (Roche Diagnostics, China) per the manufacturer's instructions, respectively. Serum ALT, creatinine (Cr), and creatine kinase (CK) were measured on an automatic biochemistry analyzer (Olympus AU5400, Japan) according to standard laboratory procedures. HBV mutations that were associated with resistance to ADV (rtA181V/T and rtN236T) and ETV (rtI169T, rtL180M, rtT184G, rtS202I, rtM204V/I, and rtM250V) were analyzed by PCR pyrosequencing assay if virological rebound (defined as a confirmed increase in HBV DNA levels by at least 1 log-copy per milliliter from the nadir value, based on PCR) occurred.

### Statistical analysis

Quantitative variables were expressed as mean values; categorical variables were expressed as counts and percentages; and HBV DNA levels were log-transformed. Comparisons between groups of quantitative and qualitative variables were analyzed by student t-test and chi-square test (or Fisher's exact test), respectively. A p-value of less than 0.05 (two-tailed) indicated a significant difference. All statistical analyses were performed using SPSS, version 13.0 (SPSS Inc., Chicago, IL).

## Results

### Characteristics of the study patients

A total of 100 patients from West China Hospital, Sichuan University were enrolled in the study between June 2007 and April 2008; 52 patients were given ADV, and 48 patients were administered ETV. In the ADV group, there were 33 HBeAg-positive patients and 19 HBeAg-negative patients at baseline. In the ETV group, there were 32 HBeAg-positive patients and 16 HBeAg-negative patients at baseline. The demographics and disease parameters in HBeAg-positive (Table 1) and HBeAg-negative (Table 1) patients were well matched between groups at baseline. No patient discontinued treatment due to poor efficacy or disease progression.

**Table 1:** Baseline characteristics of HBeAg positive and negative patients

Characteristic	ADV a		ETV b		p-value	
	Positive (N=33)	Negative (N=19)	Positive (N=32)	Negatoive (N=19)	Positive	Negatoive
Male No. (%)	24 (72.7)	13 (68.4)	23 (71.9)	14 (87.5)	0.939	0.244
Age (Mean±SD, year)	34.18	36.58	36.81	39.44	0.108	0.313
BMI (kg/m(2))	22.58	21.35	22.24	23.25	0.687	0.096
ALT (IU/L)	174.73	160.0	208.75	159.3	0.247	0.988
HBV DNA (log10 copies/ml)	7.91	6.44	7.98	6.76	0.835	0.575
Smoke No. (%)	13 (39.4)	6 (31.6)	10 (31.3)	4 (25.0)	0.492	0.723
Drink No. (%)	13 (39.4)	5 (26.3)	13 (40.6)	4 (25.0)	0.919	1.000

^a^ ADV: adefovir

^b^ ETV: entecavir

### Biochemical response

**Figure 1:**
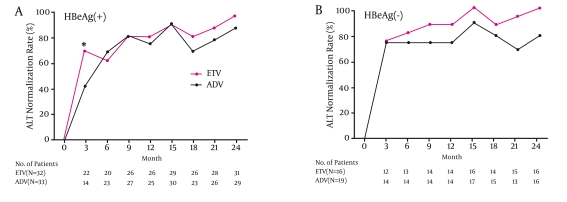
Serum ALT normalization in HBeAg-positive and HBeAg-negative patients from baseline to Month 24. Panel A- rate of ALT normalization in HBeAg-positive patients receiving ETV or ADV; the difference in ALT normalization was not significant between groups from Months 6 to 24 after treatment. Panel B- rate of ALT normalization in HBeAg-negative patients receiving ETV or ADV; the difference in ALT normalization was not significant between groups

Serum ALT levels declined in both treatment groups. At 6, 12, and 24 months, normal ALT was achieved in 23 of 33 (69.7%), 25 of 33 (75.8%), and 29 of 33 (87.9%) HBeAg-positive patients in the ADV and in 20 of 32 (62.5%), 26 of 32 (81.3%), and 31 of 32 (96.9%) HBeAg-positive patients in the ETV group, respectively (Figure 1A). In HBeAg-negative patients at 6, 12, and 24 months, normal ALT was attained in 14 of 19 (73.7%), 14 of 19 (73.7%), and 15 of 19 (78.9%) ADV-treated cases and in 13 of 16 (81.3%), 14 of 16 (87.5%), and 16 of 16 (100%) patients who were given ETV, respectively (Figure 1B). The difference in ALT normalization between groups was not significant in HBeAg-positive or HBeAg negative patients.

### Virological response

**Figure 2:**
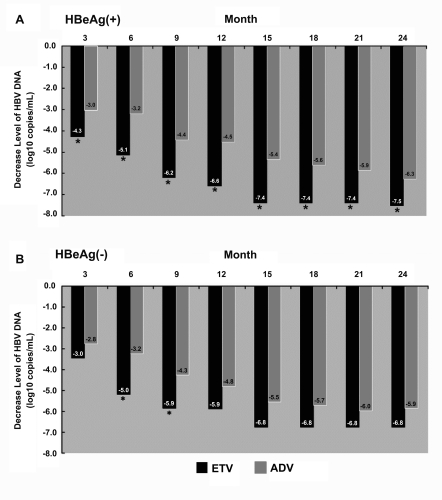
Serum reduction in HBV DNA levels from baseline. Panel A shows the reduction in HBV DNA levels from baseline to Month 24 in HBeAg-positive patients. Panel B shows the reduction in HBV DNA levels from baseline to Month 24 in HBeAg-negative patients.

**Figure 3:**
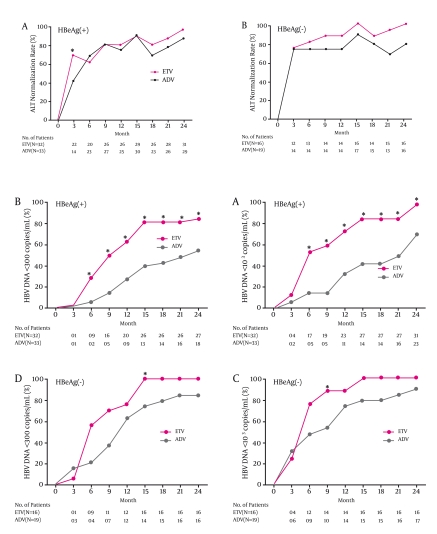
The percentage of those who obtained a virological response between ADV- and ETV-treated HBeAg-positive and HBeAg-negative patients.

In HBeAg-positive patients, the reduction in serum HBV DNA levels from baseline during the observation period was significantly greater with ETV compared with ADV (Figure 2A); at 24 months, serum HBV DNA levels declined by a mean of 5.6 and 6.0 log10 copies?mL with ADV and ETV, respectively (P=0.008). At 6, 12, and 24 months, HBV DNA < 103 copies/ml was achieved in 5 of 33 (15.2%), 11 of 33 (33.3%), and 23 of 33 (69.7%) patients with ADV and in 17 of 32 (53.1%), 23 of 32 (71.9%), and 31 of 32 (96.9%) patients with ETV, respectively (P = 0.001, P = 0.002, P = 0.003, respectively , between treatment groups) (Figure 3A). At 6, 12, and 24 months, HBV DNA < 300 copies/ml was achieved in 2 of 33 (6.1%), 9 of 33 (27.3%), and 18 of 33 (54.5%) patients with ADV and in 9 of 32 (28.1%), 20 of 32 (62.5%), and 27 of 32 (84.4%) patients with ETV, respectively (P = 0.018, P = 0.004, P = 0.009, respectively, between treatment groups) (Figure 3B).In HBeAg-negative patients, the reduction in serum HBV DNA levels from baseline did not differ between ADV and ETV treatments (Figure 2B); at 24 months, serum HBV DNA levels fell by a mean of 5.9 and 6.8 log10 copies?mL with ADV and ETV, respectively (P=0.192). At 6, 12 and 24 months, HBV DNA < 103 copies/ml was achieved in 9 of 19 (47.4%), 14 of 19 (73.7%), and 17 of 19 (89.5%) patients with ADV and in 12 of 16 (75%), 14 of 16 (87.5%), and 16 of 16 (100%) patients with ETV, respectively (P = 0.096, P = 0.309, P = 0.489, respectively, between treatment groups) (Figure 3C). At 6, 12, and 24 months, HBV DNA < 300 copies/ml was achieved in 4 of 19 (21.1%), 12 of 19 (63.2%), and 16 of 19 (84.2%) patients with ADV and in 9 of 16 (56.3%), 12 of 16 (75%), and 16 of 16 (100%) patients with ETV, respectively (P = 0.032, P = 0.452, and P = 0.234, respectively, between treatment groups) (Figure 3D).

### HBeAg response

**Figure 4:**
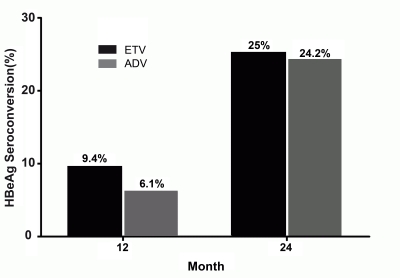
Percentage of HBeAg-positive patients with HBeAg seroconversion in the ADV and ETV groups. The differences between groups were not significant at Month 12 or 24.

Two of 33 (6.1%) and 3 of 32 (9.4%) HBeAg-positive patients experienced HBeAg seroconversion at Month 12 with ADV and ETV, respectively (P=0.672 between groups) (Figure 4). The overall HBeAg seroconversion rates in the groups continued to rise during the study period. At Month 24, the cumulative rate of HBeAg seroconversion occurred in 8 of 33 (24.2%) patients with ADV compared with 8 of 32 (25%) patients with ETV (P=0.943).

### Breakthrough and resistance

Viral breakthrough was not observed in any patients during the 2-year observation period, and HBV mutations were not analyzed in the first 2 years of treatment.

### Safety

Four patients who received ADV had slightly increased serum creatinine levels from baseline (less than 44.2 µmol per liter), but there were no discontinuations due to this adverse event. In ETV recipients, no benign or malignant lesions in the lung were detected. Elevations in ALT were observed less frequently in the ETV group than in the ADV group. ALT flares were observed in 3 patients who received ETV and 10 ADV-treated patients. In the ETV group, all ALT flares in the 2 year treatment period were associated with alcohol consumption. In the ADV group, 6 of the ALT flares were associated with alcohol consumption and 4 were associated with HBV DNA flares. In the ETV group, 1 patient developed a small hepatocellular carcinoma at Month 15 and received immediate surgical intervention; ETV treatment was never discontinued.

## Discussion

The treatment options for CHB are limited. The principal goals of anti-HBV treatment are to suppress HBV DNA, normalize ALT levels, and reduce liver necroinflammation. In this study, both ADV and ETV were efficacious in among positive- and negative-HBeAg CHB patients with regard to biochemical and virological responses. In HBeAg-positive patients, ETV was significantly superior to ADV in terms of virological response, which is consistent with other studies of ETV that have demonstrated reduced HBV DNA levels [17][18][19][20], attributed to its suppression of HBV replication.Based on the nephrotoxicity, the dose of ADV was adjusted from 30 mg to 10 mg for CHB patients, but Vitro, this dose adjustment weakened the antiviral activity of ADV, and clinical studies have reported that the virological response of ADV is less robust than that of other anti-HBV agents. In this study, the virological response of ADV in HBeAg-positive patients was significantly lower than that of ETV, but in HBeAg-negative patients, ADV had similar biochemical and virological responses as ETV.In a separate analysis, the percentage of ADV-treated HBeAg-positive and HBeAg-negative patients with HBV <300 copies/mL was 54.5% and 84.2%, respectively. This result suggests that the efficacy of ADV differs in HBeAg-negative and HBeAg-positive patients. Further, other nucleoside analogs have had high therapeutic efficacy in HBeAg-negative patients [21][22][23].But, the exact mechanism that underlies the effect of baseline negative HBeAg status on virological response remains unknown. In the ADV group, the mean baseline HBV DNA level was 6.44 and 7.91 log10 copies/mL in HBeAg-negative and HBeAg-positive patients, respectively; thus, lower baseline HBV DNA levels may contribute to a more robust virological response for ADV in HBeAg-negative patients. Some studies have suggested that baseline HBV DNA level is an independent predictor of long-term virological response [24][25].In this study, genotypic substitutions in polymerase-reverse transcriptase were detected only in patients with viral breakthrough. During the limited 2-year observation period, no viral breakthrough was observe in ADV- or ETV-treated patients; thus, the rates of viral breakthrough and resistance with ADV that we observed are better than those of other reports [26][27]. However, resistance patterns with long-term treatment were not determined in this study, and the resistance rate to ADV might increase significantly during subsequent treatments. Thus, we will continue to monitor all of our patients closely.HBeAg loss or seroconversion indicates durable immune control of hepatitis B virus. But, in this study, the percentage of patients who experienced seroconversion of HBeAg was less than 30% in both groups. Due to the absence of HBeAg loss or seroconversion, long-term treatment is often required to maintain viral suppression, and the treatment strategy for such patients should be optimized. Substituting or adding an antiviral agent that effects higher seroconversion rates is a warranted option. The data on adverse events are consistent with previous findings. Severe renal impairment was not observed in patients who received ADV. Further, 4 of the 52 ADV recipients had slightly increased serum creatinine levels from baseline; they were monitored closely, and the levels normalized without any additional treatment. In this study, flares of ALT were more frequent in patients who consumed alcohol. Health education must be stressed for patients with poor compliance. After reviewing the family history of a patient who developed HCC in the ETV group, we noted that his father and brother died from HBV-associated HCC. Thus, patients with a family history of HCC should be monitored closely.In this study, many observations were difficult to explain. For example, why were the dynamics of the viral response similar in only some ADV-treated and ETV-treated patients? Alterations in drug absorption and metabolism of ADV in patients should be considered. One could argue that these patients, with HBV DNA> 103 copies/mL, simply need an adjustment of treatment strategies to overcome the weakened responsiveness, such as switching to or adding another potent anti-HBV agent that does not have crossresistance to ADV. Our study complements the antiviral data on nucleoside and nucleotide analogs. ETV should be recommended for both HBeAg-positive and HBeAg-negative patients. However, for naïve chronic hepatitis B patients who are HBeAg-negative and have lower HBV DNA levels at baseline, ADV should also be considered. Adefovir and entecavir were effective as CHB treatments. In HBeAg-positive patients, the efficacy of ETV was significantly superior to that of ADV, but similar biochemical and virological responses were observed with both agents in HBeAg-negative patients. Thus, adefovir can be considered for HBeAg-negative patients with lower HBV DNA levels at baseline.
